# Infant feeding practices and autism spectrum disorder in US children aged 2–5 years: the national survey of children’s health (NSCH) 2016–2020

**DOI:** 10.1186/s13006-023-00580-2

**Published:** 2023-08-11

**Authors:** Xiao-Ling Zhan, Ning Pan, Shamshad Karatela, Lei Shi, Xin Wang, Zhao-Yan Liu, Jin Jing, Xiu-Hong Li, Li Cai, Li-Zi Lin

**Affiliations:** 1 Research Center of Children and Adolescent Psychological and Behavioral Development, Department of Maternal and Child Health, School of Public Health, Sun Yat-sen University, 510080 Guangzhou, China; 2 Key Laboratory of Brain, Cognition and Education Sciences, Institute for Brain Research and Rehabilitation, Ministry of Education, South China Normal University, 510631 Guangzhou, China; 3 Faculty of Health and Behavioural Sciences, Pharmacy Australia Centre of Excellence, University of Queensland, Woolloongabba, QLD Australia; 4 Institute of Tropical Health and Medicine (AITHM), James Cook University, Townsville, QLD Australia; 5 JNU-HKUST Joint Laboratory for Neuroscience and Innovative Drug Research, College of Pharmacy, Jinan University, Guangzhou, 510632 Guangdong China; 6 Department of Nutrition, School of Public Health, Sun Yat-sen University, 510080 Guangzhou, China; 7 Guangdong Provincial Engineering Technology Research Center of Environmental Pollution and Health Risk Assessment, Department of Occupational and Environmental Health, School of Public Health, Sun Yat-sen University, 74 Zhongshan 2nd Road, Yuexiu District, 510080 Guangzhou, China

**Keywords:** Autism spectrum disorder, Feeding behavior, Cross-sectional studies, United States, Risk factors

## Abstract

**Objective:**

To investigate the relationship between infant feeding practices and autism spectrum disorder (ASD) among children aged 2–5 years in the United States (US).

**Methods:**

Data from the 2016–2020 National Survey of Children’s Health, a nationally representative cross-sectional survey, were utilized for this study. Questionnaires were administered to parents of children aged 2–5 years to gather information on ASD diagnosis, infant feeding practices, and demographic factors (e.g., child sex, ethnic group, and maternal age at birth). Logistic regression with sample weights was employed to assess the association between infant feeding practices and ASD, while controlling for demographic variables. Polynomial regression models were used to examine trends in exclusive breastfeeding and ever breastfeeding rates among children with and without ASD.

**Results:**

A total of 35,050 children aged 2–5 years were analyzed, including 616 diagnosed with ASD, after excluding participants with missing information on breastfeeding and ASD diagnosis. Of these children with ASD, 76.6% (n = 472) had a breastfeeding history, with 66.6% (n = 410) engaged in partial breastfeeding and 10.1% (n = 62) exclusively breastfed. Adjusted odds ratios for each additional month of breastfeeding compared to never being breastfed were 0.99 (95% CI, 0.97–1.01). The adjusted odds ratios for breastfeeding durations of > 0–6 months, > 6–12 months, > 12–24 months, and > 24 months were 0.84 (95% CI, 0.51–1.36), 0.76 (95% CI, 0.42–1.35), 0.79 (95% CI, 0.43–1.45), and 0.66 (95% CI, 0.32–1.35), respectively. Compared to children who were never breastfed, the adjusted odds ratio for children who were ever breastfed was 0.79 (95% CI, 0.50–1.25). Among children with ASD, the proportion of ever breastfeeding declined from 82.0% in 2017 to 64.3% in 2020, while exclusive breastfeeding decreased from 12.0% in 2016 to 5.9% in 2020.

**Conclusions and relevance:**

Although no significant association was found between infant feeding practices and ASD among US children aged 2–5 years, the rates of breastfeeding, particularly exclusive breastfeeding, were suboptimal among children with ASD. This highlights the need for specific policies and practices to promote and support breastfeeding among parents of children with ASD or those at high risk of having a child with ASD.

**Supplementary Information:**

The online version contains supplementary material available at 10.1186/s13006-023-00580-2.

## Introduction

Breastfeeding practices play an important role in the maturation of neural systems and the development of the offspring’s social behaviors [1]. However, considerable controversy exists over the infant feeding practices on the development of autism spectrum disorder (ASD) [2], a group of heterogeneous, early-onset developmental disorders characterized by core deficits in social communication and the presence of restricted, stereotypical patterns of behaviors, interests, or activities [3]. Approximately 1 in 100 children globally are estimated to have ASD [4]. In addition, the disability-adjusted life years (DALYs) attributed to ASD have increased from 3.91 million (2009) to 4.31 million (2019), accounting for substantial health loss across the lifespan [5]. Early intervention is essential, but the strength of evidence for most interventions remains unclear [6]. Identifying modifiable factors like breastfeeding practices in early life is required to tailor prevention strategies and practice implications in order to reduce the incidence of ASD.

Breast milk contains bioactive factors, hormones, and growth factors that support the maturation of the infant’s brain [7]. These include gangliosides, phospholipids, and sialic acid, which have been suggested to have a potential link with the development of ASD [8–10]. However, current epidemiological evidence regarding the association between infant feeding practices and ASD is inconsistent as highlighted in Supplemental Table S1. Among the 26 previous studies investigating the relationship between breastfeeding and ASD, nine found no significant association, while 15 reported a reduced risk of ASD associated with breastfeeding and two indicated the opposite effect. These inconsistent findings can be attributed to the limited availability of detailed information concerning infant feeding practices. As an illustration, the investigation of dose-response associations through the utilization of breastfeeding duration was undertaken in four studies [11–14], while exclusive breastfeeding and/or partial breastfeeding information was provided in only nine studies [12, 13, 15–21]. Furthermore, although breastfeeding practices have been associated with long-term wellbeing in children [22], it is noteworthy that women who are autistic or exhibit autistic traits may possess a heightened risk factor for giving birth to children with ASD [23]. These women may also encounter additional obstacles when it comes to breastfeeding, as compared to neurotypical women [24]. Nevertheless, our analysis revealed that only three studies have reported breastfeeding rates specifically among children with ASD [25–27].

Hence, the objective of this study was to thoroughly examine the relationship between breastfeeding and ASD by utilizing the National Survey of Children’s Health (NSCH), a national population-based study in the United States (US). For this study, we merged five cycles of data from NSCH spanning 2016 to 2020 in order to explore the association. Our hypothesis posited that infant feeding practices might be linked to the likelihood of ASD among children aged 2 to 5 years. Furthermore, we anticipate observing disparities in breastfeeding practices between children with and without ASD.

## Methods

### Study population

Data for this study were obtained from the NSCH conducted between 2016 and 2020. The NSCH is a cross-sectional survey designed to capture information on the welfare of children aged 0–17 years in the US. The survey encompasses non-institutionalized children who live with their families or guardians and excludes those residing in institutional settings like orphanages or group homes. The funding and oversight for the NSCH are provided by the Health Resources and Services Administration Maternal and Child Health Bureau [28]. Ethical approval for all survey procedures is obtained from the National Center for Health Statistics Research Ethics Review Board. Written consent is obtained through electronic submission or paper mailing, and it is returned to the US Census Bureau [29]. This study of an anonymous public dataset with no identifiable information on the survey participants was determined exempt by the Institutional Review Board of the Sun Yat-sen University. The NSCH data collection encompasses all 50 states and the District of Columbia, providing a comprehensive representative of the population in the US [30, 31].

To ensure accurate estimation of population parameters by accounting for clustering, the NSCH employs a complex survey design. Clustering arises when individuals within a group share similar characteristics, and failure to address it can introduce bias in estimates. To address this, the NSCH adopts a multistage probability sample design. First, primary sampling units (PSUs), consisting of counties or groups of counties are selected. Then, households within each PSU are chosen, and finally, one child is selected within each household. To encourage participation, the NSCH provides a monetary incentive of $2 or $5 for randomly selected individuals who complete the survey. Respondents are given the option to complete the survey either online or using paper forms. The NSCH emphasizes consistency in its data collection methodology and variable coding across different survey years, allowing for the merging of data from multiple years, such as the combination of five cycles from 2016 to 2020. The NSCH survey methodology report [30, 32] and documents of data users frequently asked questions [33] offer a comprehensive description of the frame and sample selection procedures, data collection methodology, and data collection results. A condensed summary of the aforementioned details is also available in the **supplemental material**.

**Fig. 1:**
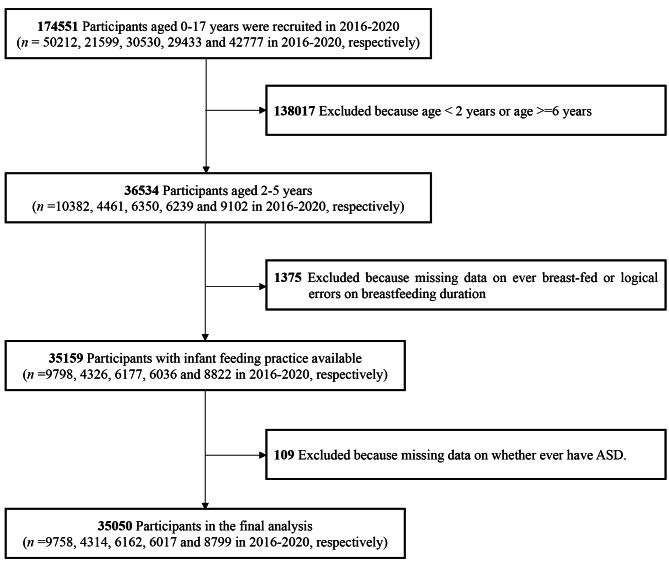
Flowchart with the final analysis of the unweighted survey sample sizes for children aged 2–5 years, National Survey of Children’s Health, 2016–2020

The study’s inclusion and exclusion criteria were established to select respondents who had children between the ages of 2 and 5 years. This age range was chosen due to its relevance to reliable early diagnosis of ASD [34], and respondents were required to have answered questions regarding both ASD diagnosis and infant feeding practices in the survey. Out of the initial sample of 36,534 children aged 2–5 years from the survey, a total of 35,050 children met the criteria for inclusion in this analysis. These children were selected based on the completeness of data for ASD diagnosis, breastfeeding duration, and other relevant demographic variables, as depicted in Fig. 1 (flowchart).

### Measurements of ASD and infant feeding practices

In the NSCH, the identification of ASD was based on parents’ affirmative responses to the question, “Has a doctor or other health care provider ever informed you that this child has autism or autism spectrum disorder (ASD)? Include diagnoses of Asperger’s Disorder or Pervasive Developmental Disorder (PDD)”. Additionally, a current diagnosis of ASD was determined by asking the caregivers, “Does this child currently have the condition?”. The measure of ever breastfeeding was obtained from the question, “Was this child ever breastfed or fed breast milk?”, with response options of yes or no. For caregivers who responded affirmatively, a follow-up question was included: “If yes, how old was this child when they completely stopped breastfeeding or being fed breast milk?”. By utilizing responses to these two items, we derived a continuous variable representing breastfeeding duration. To facilitate analysis, we further categorized breastfeeding duration into five distinct categories: never, > 0–6 months, > 6–12 months, > 12–24 months, and > 24 months. Based on responses to the questions “How old was this child when they were first fed formula?“ and “How old was this child when they were first fed anything other than breast milk or formula?”, the breastfeeding status was determined and categorized into three distinct groups: [1] no breastfeeding, [2] partial breastfeeding (which includes the introduction of other liquids or solids alongside breast milk for infants below 6 months of age), and [3] exclusive breastfeeding (indicating that the infant received only breast milk and no other liquids or solids during the first 6 months of life) [35].

### Covariates

Demographic data encompassing child sex, child ethnic group, the highest educational level in the household, maternal age at birth, family income, low birth weight, preterm birth, and birth order were collected using a questionnaire. Child ethnic group was classified into distinct categories, including white alone, black or African American alone, American Indian or Alaska native alone, and others, which encompassed Asian alone, native Hawaiian and other Pacific islander alone, some other race alone, and two or more races. The highest educational level in the household was represented as a categorical variable with options including less than a high school education, high school education, some college or associate degree, and college degree or higher. Maternal age at birth was categorized into three groups: ≤25 years, > 25–35 years, and > 35 years. Family income, expressed as a percentage of the federal poverty level (FPL), was divided into four categories: <100%, 100–199%, 200–399%, and ≥ 400%, based on the 2010 FPL. Other variables including low birth weight, preterm birth, and only child, were dichotomized as yes or no.

### Statistical analysis

Differences in demographic characteristics and infant feeding practices between children with and without ASD using chi-square tests. Logistic regression models were employed to assess the association between infant feeding practices and ASD. Initially, crude models were fitted without any adjustment, followed by adjusted models that controlled for child age, child sex, year of birth, ethnic group, family income, and birth order. The selection of covariates was based on collinearity tests. Subgroup analyses were conducted based on the child’s sex (male vs. female) to estimate the associations of infant feeding practices with ASD, considering that the sex of the child has been found to influence breastfeeding initiation and duration in certain countries [36]. The analyses conducted in this study utilized the sampling weights provided in the public-use NSCH data set. These weights consist of a base sampling weight and adjustments for factors such as multiple telephone lines per household and non-response [37–39]. We also performed additional analyses without applying survey weights. Sensitivity analysis was conducted to examine the association between children with a current diagnosis of ASD and infant feeding practices. To assess the trends in exclusive breastfeeding and ever breastfeeding across different groups (children with and without ASD) using multiple years of NSCH data from 2016 to 2020, linear trend analysis was conducted using the “segmented” package (Vito M. R. Muggeo) [40] and the “survey” package (Thomas Lumley) [41].

In all analysis procedures, we employed survey weights, strata, and primary sampling units provided along with the NSCH data to ensure the representation of noninstitutionalized children in the US. The use of survey weighting in the NSCH guarantees a representative sample of the population of interest, and the inclusion of survey-weighted analysis is crucial for accurate estimation and interpretation of the results.

All statistical analyses were performed using the R Core Team Statistical Software, version 4.1.0 (2021), and two-sided *P*-values were calculated. Statistical significance was considered at a *P*-value less than 0.05.

## Results

A total of 36,534 participants aged 2 to 5 years were enrolled between 2016 and 2020. Exclusions were made for participants with missing information on breastfeeding (n = 1375) or autism spectrum disorder diagnosis (n = 109). Consequently, the final analysis included 35,050 children aged 2 to 5 years (9758, 4314, 6162, 6017 and 8799 in 2016–2020, respectively), comprising 34,434 children without ASD and 616 children with a history of ASD diagnosis. The weighted prevalence of ASD in this study was determined to be 1.9% (unweighted 1.8%) among children aged 2 to 5 years in the US, which is higher than the worldwide prevalence of autism, which is approximately 1 in 100 individuals [4]. We compared the differences between the two groups (children with and without ASD) in demographic characteristics and breastfeeding practices and the results are presented in Table 1. Among the 616 children with ASD, the average age was 3.69 ± 0.10 years, with a majority being male (75.4%) and 57.9% classified as white. Of these children, 76.6% (472 ASD children) had a history of breastfeeding, with 66.6% engaging in partial breastfeeding and 10.1% being exclusively breastfed. In comparison to children without ASD, families of children with ASD exhibited lower socioeconomic status, as evidenced by their lower highest educational level in the household and family income (*P* < 0.001). Furthermore, children with ASD had higher rates of preterm birth and were more likely to be only children, when compared to children without ASD (*P* < 0.05).

**Table 1:** Descriptive statistics of participant characteristics _a_

	**Without ASD** **(N = 34434)**		**With ASD** (N = 616)		*t value/F*	*df*	*P* value
	Unweighted sample size (unweighted%)	Weighted% (95%*CI*)	Unweighted sample size (unweighted%)	Weighted% (95%*CI*)			
**Child Age, y**	3.46 ± 1.13	3.48 ± 0.01	3.76 ± 1.03	3.69 ± 0.10	1.94	34998	0.05
**Child Sex**					31.02	34999	**< 0.001**
Male	17674 (51.33%)	50.6% (49.4% − 52.0%)	484 (78.57%)	75.4% (66.3% − 83.0%)			
Female	16760 (48.67%)	49.4% (48.2% − 51.0%)	132 (21.43%)	24.6% (17.4% − 34.0%)			
**Ethnic group**					5.15	34997	**< 0.01**
White	26539 (77.07%)	67.7% (66.5% − 69.0%)	429 (69.64%)	57.9% (48.8% − 66.0%)			
Black or American	2065 (6.00%)	12.6% (11.7% − 13.0%)	61 (9.90%)	20.5% (13.5% − 30.0%)			
American Indian or Alaska native	263 (0.76%)	1.6% (1.2% − 2.0%)	5 (0.81%)	0.3% (0.1% − 1.0%)			
Other	5567 (16.17%)	18.1% (17.1% − 19.0%)	121 (19.64%)	21.3% (14.8% − 30.0%)			
**Highest educational level in the household** ^**b**^					7.47	34996	**< 0.001**
Less than high school	602 (1.75%)	6.4% (5.6% − 7.0%)	22 (3.57%)	10.5% (6.0% − 18.0%)			
High school	3703 (10.75%)	18.2% (17.1% − 19.0%)	95 (15.42%)	25.8% (18.2% − 35.0%)			
Some college or Associate Degree	7195 (20.90%)	21.2% (20.3% − 22.0%)	171 (27.76%)	30.5% (22.6% − 40.0%)			
College degree or higher	22849 (66.36%)	53.9% (52.7% − 55.0%)	328 (53.25%)	33.2% (26.8% − 40.0%)			
**Maternal age when giving birth** ^**c**^					0.48	34997	0.70
≤ 25 years	6625 (19.24%)	23.2% (22.2% − 24.0%)	132 (21.43%)	24.7% (17.1% − 34.0%)			
> 25–35 years	21600 (62.73%)	58.0% (56.8% − 59.0%)	359 (58.28%)	56.9% (48.0% − 65.0%)			
> 35 years	5803 (16.85%)	17.2% (16.3% − 18.0%)	116 (18.83%)	15.2% (10.8% − 21.0%)			
**Family income, % FPL**					6.07	34997	**< 0.001**
0–99	3275 (9.51%)	17.3% (16.2% − 18.0%)	109 (17.69%)	26.4% (19.5% − 35.0%)			
100–199	5666 (16.45%)	22.4% (21.3% − 24.0%)	136 (22.08%)	33.9% (25.1% − 44.0%)			
200–399	12170 (35.34%)	32.1% (31.0% − 33.0%)	210 (34.09%)	22.4% (17.3% − 28.0%)			
≥ 400	13323 (38.69%)	28.2% (27.3% − 29.0%)	161 (26.14%)	17.3% (13.0% − 23.0%)			
**Low birth weight** ^**d**^					1.98	34998	0.14
Yes	2672 (7.76%)	8.7% (8.0% − 9.0%)	80 (12.99%)	14.6% (9.7% − 22.0%)			
No	30849 (89.59%)	87.7% (86.8% − 88.0%)	520 (84.42%)	82.2% (75.0% − 88.0%)			
**Preterm birth** ^**e**^					3.83	34998	**0.02**
Yes	3481 (10.11%)	11.1% (10.3% − 12.0%)	110 (17.86%)	18.1% (13.3% − 24.0%)			
No	30718 (89.21%)	88.1% (87.2% − 89.0%)	498 (80.84%)	79.9% (73.4% − 85.0%)			
**Only child**					4.44	34999	**0.04**
Yes	22326 (64.84%)	21.8% (21.0% − 23.0%)	359 (58.28%)	30.7% (23.3% − 39.0%)			
No	12108 (35.16%)	78.2% (77.3% − 79.0%)	257 (41.72%)	69.3% (60.7% − 77.0%)			
**Breastfeeding Duration in Categories**					1.70	34996	0.15
Never	5665 (16.82%)	21.5% (20.4% − 23.0%)	144 (23.68%)	31.0% (22.7% − 41.0%)			
> 0–6 months	11609 (34.46%)	34.7% (33.5% − 36.0%)	241 (39.64%)	35.7% (28.2% − 44.0%)			
> 6–12 months	8473 (25.15%)	22.6% (21.6% − 24.0%)	102 (16.78%)	17.0% (11.7% − 24.0%)			
> 12–24 months	6573 (19.51%)	17.7% (16.8% − 19.0%)	96 (15.79%)	13.7% (9.2% − 20.0%)			
> 24 months	1370 (4.07%)	3.6% (3.2% − 4.0%)	25 (4.11%)	2.6% (1.4% − 5.0%)			
**Ever Breast-fed**					3.81	34999	0.05
No	5665 (16.45%)	21.1% (20.0% − 22.0%)	144 (23.38%)	30.8% (22.5% − 41.0%)			
Yes	28769 (83.55%)	78.9% (77.9% − 80.0%)	472 (76.62%)	69.2% (59.4% − 77.0%)			
**Breastfeeding Status**					2.70	34998	0.07
No Breastfeeding	5665 (16.45%)	21.1% (20.0% − 22.0%)	144 (23.38%)	30.8% (22.5% − 41.0%)			
Partial Breastfeeding	25425 (73.84%)	69.0% (67.9% − 70.0%)	410 (66.56%)	57.3% (48.2% − 66.0%)			
Exclusive Breastfeeding	3344 (9.71%)	9.9% (9.2% − 11.0%)	62 (10.06%)	11.8% (7.3% − 19.0%)			

^a^ Data are from the 2016–2020 National Survey of Children’s Health (N = 35,050)

^b^ Data were missing for 85 children without ASD.

^c^ Data were missing for 406 children without ASD and 9 children with ASD.

^d^ low birth weight was defined as birth weight < 2500 g; Data were missing for 913 children without ASD and 16 children with ASD.

^e^ Preterm birth was defined as born 3 weeks before due date; Data were missing for 235 children without ASD and 8 children with ASD.

As presented in Table 2, the previously observed negative association between infant feeding practices and ASD among children did not remain statistically significant after adjusting for covariates. The adjusted odds ratio (OR) for each additional month of breastfeeding was 0.99 (95% CI, 0.97–1.01). When comparing children who never breastfed to those with varying durations of breastfeeding, the adjusted ORs for children breastfed for 0–6 months, 6–12 months, 12–24 months, and longer than 24 months were 0.84 (95% CI, 0.51–1.36), 0.76 (95% CI, 0.42–1.35), 0.79 (95% CI, 0.43–1.45), and 0.66 (95% CI, 0.32–1.35), respectively. Furthermore, compared with children who were never breastfed, the adjusted OR for children who were ever breastfed was 0.79 (95% CI, 0.50–1.25). When examining exclusive breastfeeding and partial breastfeeding in relation to no breastfeeding, the adjusted ORs ranged from 1.12 (95% CI, 0.57–2.20) for exclusive breastfeeding to 0.74 (95% CI, 0.47–1.18) for partial breastfeeding. As Table 3 shows, each additional month of breastfeeding was associated with decreased risk of ASD (OR 0.98, 95% CI, 0.97–1.00) in the unweighted analyses. When comparing children who never breastfed, the adjusted ORs for children breastfed for 6–12 months, ever breastfed and partial breastfeeding were 0.63 (95% CI, 0.48–0.83), 0.81 (95% CI, 0.67–1.00) and 0.80 (95% CI, 0.65–0.98) in the unweighted analyses, respectively. We found similarly null associations between current ASD and breastfeeding when considering the weighted analysis (Data shown in Table 4).

**Table 2:** Associations of infant feeding practices with ASD among US children aged 2–5 years (weighted data)

	Crude Model					Adjusted Model			
	*t*	df	ORs (95% CI)	*P*		*t*	df	aORs (95% CI)	*P*
**Breastfeeding Duration, Months**	-2.30	34246	0.97 (0.95, 1.00)	**0.02**		-0.90	34226	0.99 (0.97, 1.01)	0.37
**Breastfeeding Duration in Categories**									
**Never**	*Reference*					*Reference*			
**> 0–6 months**	-1.41	34243	0.71 (0.45, 1.14)	0.16		-0.72	34223	0.84 (0.51, 1.36)	0.47
**> 6–12 months**	-2.31	34243	0.52 (0.30, 0.91)	**0.02**		-0.94	34223	0.76 (0.42, 1.35)	0.35
**> 12–24 months**	-2.16	34243	0.54 (0.30, 0.95)	**0.03**		-0.76	34223	0.79 (0.43, 1.45)	0.45
**> 24 months**	-1.97	34243	0.49 (0.24, 1.00)	**0.05**		-1.14	34223	0.66 (0.32, 1.35)	0.26
**Ever Breast-fed**									
**No**	*Reference*					*Reference*			
**Yes**	-2.33	34998	0.60 (0.39, 0.92)	**0.02**		-1.01	34978	0.79 (0.50, 1.25)	0.31
**Breastfeeding Status**									
**No Breastfeeding**	*Reference*					*Reference*			
**Partial Breastfeeding**	-2.54	34997	0.57 (0.37, 0.88)	**0.01**		-1.26	34977	0.74 (0.47, 1.18)	0.21
**Exclusive Breastfeeding**	-0.63	34997	0.82 (0.43, 1.54)	0.53		0.34	34977	1.12 (0.57, 2.20)	0.74

Adjusted models were adjusted for child age, child sex, year of birth, ethnic group, family income and birth order

**Table 3:** Associations of infant feeding practices with ASD among US children aged 2–5 years (unweighted data)

	Crude Model					Adjusted Model			
	*t*	df	ORs (95% CI)	*P*		*t*	df	aORs (95% CI)	*P*
**Breastfeeding Duration, Months**	-4.36	34296	0.97 (0.96,0.98)	**< 0.01**		-2.67	34275	0.98 (0.97, 1.00)	**0.01**
**Breastfeeding Duration in Categories**									
**Never**	*Reference*					*Reference*			
**> 0–6 months**	-1.90	34293	0.82 (0.66, 1.01)	**0.06**		-0.59	34272	0.94 (0.75, 1.16)	0.56
**> 6–12 months**	-5.73	34293	0.47 (0.37, 0.61)	**< 0.01**		-3.30	34272	0.63 (0.48, 0.83)	**< 0.01**
**> 12–24 months**	-4.17	34293	0.57 (0.44, 0.75)	**< 0.01**		-1.93	34272	0.76 (0.58, 1.00)	0.05
**> 24 months**	-1.52	34293	0.72 (0.47, 1.10)	0.13		-0.94	34272	0.81 (0.52, 1.26)	0.35
**Ever Breast-fed**									
**No**	*Reference*					*Reference*			
**Yes**	-4.55	35048	0.65 (0.53, 0.78)	**< 0.01**		-2.00	35027	0.81 (0.67, 1.00)	**0.05**
**Breastfeeding Status**									
**No Breastfeeding**	*Reference*					*Reference*			
**Partial Breastfeeding**	-4.64	35047	0.63 (0.52, 0.77)	**< 0.01**		-2.13	35026	0.80 (0.65, 0.98)	**0.03**
**Exclusive Breastfeeding**	-2.06	35047	0.73 (0.54, 0.99)	**0.04**		-0.50	35026	0.92 (0.68, 1.26)	0.62

Adjusted models were adjusted for child age, child sex, year of birth, ethnic group, family income and birth order

**Table 4:** Associations of infant feeding practices with current ASD among US children aged 2–5 years (weighted data)

	Crude Model					Adjusted Model			
	*t*	df	ORs (95% CI)	*P*		*t*	df	aORs (95% CI)	*P*
**Breastfeeding Duration, Months**	-2.21	34,240	0.97 (0.95, 1.00)	**0.03**		-0.80	34220	0.99 (0.97, 1.01)	0.43
**Breastfeeding Duration in Categories**									
**Never**	*Reference*					*Reference*			
**> 0–6 months**	-1.10	34237	0.77 (0.48, 1.23)	0.27		-0.39	34217	0.91 (0.55, 1.49)	0.70
**> 6–12 months**	-1.99	34237	0.57 (0.33, 0.99)	**0.05**		-0.56	34217	0.84 (0.46, 1.53)	0.57
**> 12–24 months**	-1.89	34237	0.58 (0.32, 1.02)	0.06		-0.48	34217	0.86 (0.45, 1.62)	0.63
**> 24 months**	-1.98	34237	0.47 (0.22, 0.99)	**0.05**		-1.16	34217	0.63 (0.29, 1.37)	0.25
**Ever Breast-fed**									
**No**	*Reference*					*Reference*			
**Yes**	-2.02	34991	0.64 (0.42, 0.99)	**0.04**		-0.64	34971	0.86 (0.54, 1.37)	0.52
**Breastfeeding Status**									
**No Breastfeeding**	*Reference*					*Reference*			
**Partial Breastfeeding**	-2.28	34990	0.60 (0.39, 0.93)	**0.02**		-0.93	34970	0.80 (0.50, 1.28)	0.35
**Exclusive Breastfeeding**	-0.27	34990	0.92 (0.49, 1.73)	0.79		0.68	34970	1.27 (0.64, 2.52)	0.50

Adjusted models were adjusted for child age, child sex, year of birth, ethnic group, family income and birth order

There was no statistical evidence to support the presence of associations between infant feeding practices and the risks of ASD stratified by sex (Data shown in Supplemental Table S2-S3).

**Fig. 2:**
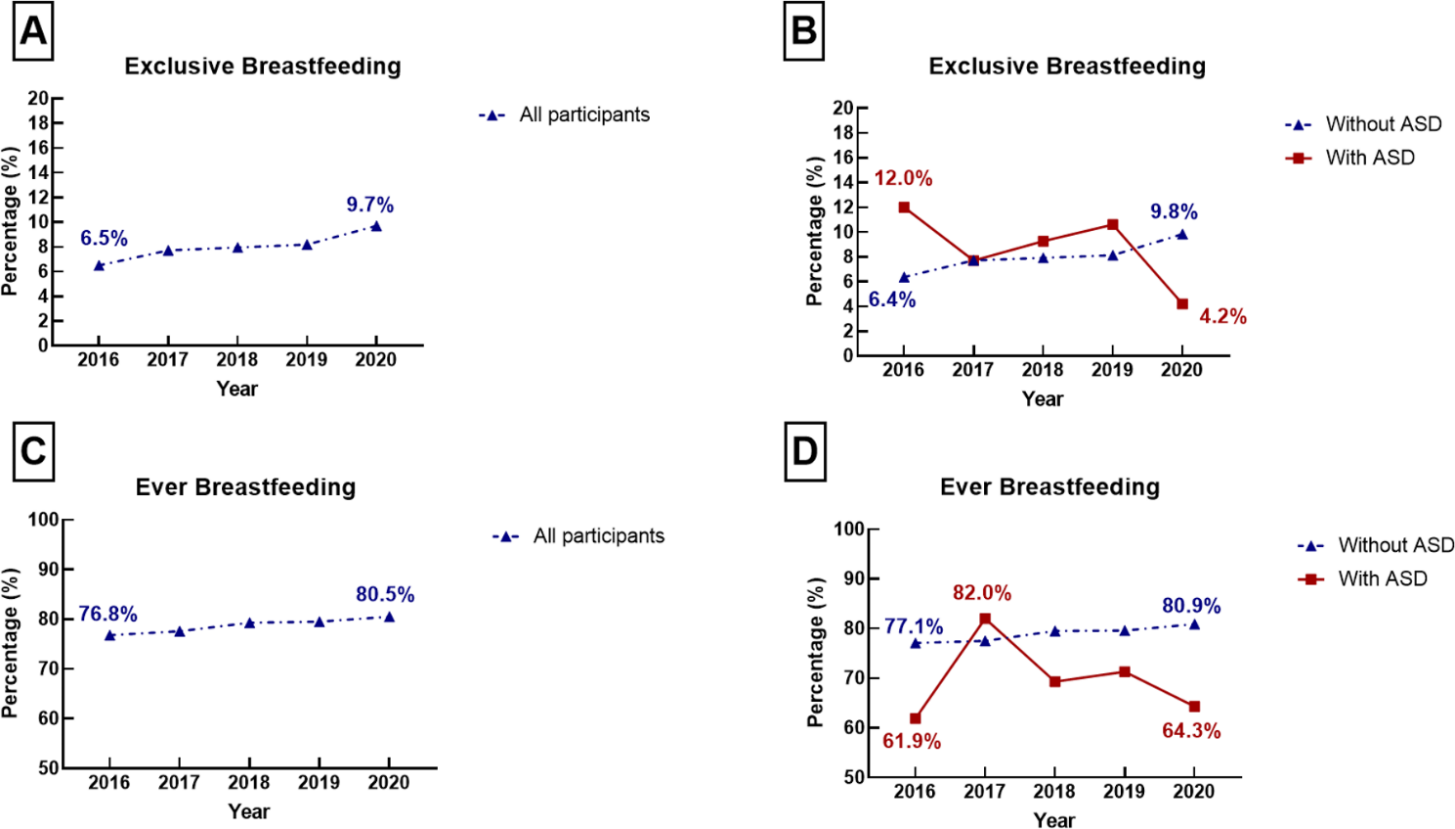
The weighted proportion of exclusive breastfeeding and ever breastfeeding among the US children with and without ASD, 2016–2020

Figure 2 illustrates the trends in the weighted proportions of breastfeeding practices, including ever breastfeeding and exclusive breastfeeding, among children with and without ASD. Among children with ASD, the weighted proportion of ever breastfeeding showed a decline from 82.0% in 2017 to 64.3% in 2020, while exclusive breastfeeding decreased from 12.0% in 2016 to 5.9% in 2020 (Data shown in Supplemental Table S4-S6).

## Discussion

In this nationwide cross-sectional study conducted in the US from 2016 to 2020, we found no significant association between infant feeding practices and the likelihood of ASD among children. However, our study revealed that breastfeeding rates among US children were significantly below the recommended levels. Specifically, we observed a decline in the weighted proportion of exclusive breastfeeding and ever breastfeeding among children with ASD over the study period.

### Association between infant feeding practices and the risk of ASD

The association between infant feeding practices and the risk of ASD has been widely investigated in previous studies. While many of these studies have reported a protective effect of breastfeeding in reducing the risk of ASD among children, our findings suggest null associations, as observed with both categorical and quantitative data on breastfeeding. We propose several interpretations for these inconsistent findings. Firstly, methodological variations in the adjustment for confounding factors between studies may be a key reason for the discrepancies in reported findings. Some studies conducted in India, Australia, Denmark, and the US have reported crude associations between breastfeeding and ASD risk without adequately accounting for potential confounders, which could introduce important biases [15, 18, 42–45]. In contrast, our findings are consistent with the majority of previous studies that have adjusted for potential confounding factors [12–14, 20, 21, 26, 46].

Secondly, there is the regional variation in the association between breastfeeding and ASD, with studies conducted in Asia predominantly reporting a protective effect of breastfeeding (7 out of 8), while studies in Western countries generally indicate null associations. Cross-cultural studies have suggested that breastfeeding practices can be influenced by cultural, ethnic, and socioeconomic factors [47, 48]. For example, in many Asian families, there is a tradition of postpartum confinement, during which mothers focus on developing breastfeeding skills [49]. This period involves rest and nursing, with mothers adhering to specific dietary restrictions and limited mobility [50]. In contrast, Western mothers may not follow the practices due to cultural, climate, dietary, and other factors, potentially resulting in different breastfeeding patterns. Thirdly, most previous studies examining the association between breastfeeding and ASD have used small sample size and limited measurements of breastfeeding practices. In contrast, our study utilized a large-scale sample and comprehensive measurements of breastfeeding practices. Our findings align with previous large-scale studies that also found no association between breastfeeding and the risk of ASD after accounting for potential confounding factors. These studies include research by Husk et al. [20], using data from 2007 to 2011 NSCH, Soke et al. [51], using data from the Study to Explore Early Development, a multi-site community-based case-control study (577 ASD and 794 controls, 30–68 months) and Dodds et al. [52] using data from a longitudinal cohort study conducted in Canada. Our nationwide cross-sectional study provides evidence of null associations between infant feeding practices and the risk of ASD among children. We have considered potential confounding factors and utilized a large sample size, making our findings robust. Furthermore, our study contributes to the existing literature by incorporating comprehensive measurements of breastfeeding practices.

### Temporal trends in the rate of infant feeding practices among children

While our study did not reveal a significant association between infant feeding practices and ASD among children, it is important to highlight the concerning rate of exclusive breastfeeding among all participants, which was observed to be only 9.7% in 2020. This finding indicates a significant deviation from the recommended rate set by the World Health Organization (WHO). WHO has reported that globally, only approximately 40% of infants under six months of age were exclusively breastfed from 2015 to 2020 and aims to increase this rate to at least 50% by 2025 [53]. Of greater significance, our study uncovered a notable trend indicating a decline in breastfeeding practices, including exclusive breastfeeding and ever breastfeeding, among mothers of children with ASD over the years. Understanding the factors contributing to this decrease in breastfeeding rates among children with ASD is crucial, and concerted efforts should be made to address this issue. Several factors may contribute to the declining breastfeeding rates among mothers of children with ASD. Although many children do not receive a definitive ASD diagnosis until later in life [54], the earliest signs of emerging ASD usually appear in the early years, such as atypical early vocal calls (i.e., infant cry) [55], diminished behavioral response to affective speech, and abnormal caregiver-child interactions [56]. These unusual signs may contribute to the declining rate of breastfeeding among mothers whose children have been diagnosed with ASD [57]. Moreover, parents of children with ASD are more likely to possess autistic traits [58] and breastfeeding present additional challenges for mothers with autistic traits compared to mothers of typically developing children [24, 59]. Breastfeeding, especially exclusive breastfeeding, remains one of the most effective strategies for preventing child infections, morbidity, and mortality during early life [60]. From a long-term perspective, breast milk contains numerous bioactive components, including immune factors, cytokines that safeguard against various infections such as ear, throat, and sinus infections [61], as well as hormones and neurotrophic factors that regulate energy intake, fostering healthy physical growth and brain development [9]. Additionally, breast milk contributes to the establishment of the microbiome, and alterations in the microbiome could impact gene expression and have lifelong implications for health and well-being, such as obesity prevention [7]. While breastfeeding is well-known for its protective effects on typical developing infants against the aforementioned short- and long-term illnesses and diseases [1], recent studies indicate an increasing prevalence of these co-occurring medical and psychiatric conditions throughout the lifespan of individuals with ASD [62]. However, the rate of breastfeeding among children with ASD is declining, underscoring the necessity for policies and practices that promote and support breastfeeding within this high-risk population.

### Implications for future research, policy, and practice

The current international breastfeeding guidelines do not sufficiently address the specific needs of parents with ASD children or high-risk parents of ASD children. Based on our findings, there are several important implications that should be carefully considered to optimize breastfeeding practices. Firstly, there is a need to enhance prenatal and/or perinatal education for parents and family members, particularly targeting high-risk parents of ASD children or parents who themselves are with autistic traits. It is crucial for these parents to receive comprehensive and tailored support to navigate the unique challenges they may face in breastfeeding. Additionally, parents, especially mothers, who are at a higher risk of having children with ASD, may benefit from specific education and guidance regarding recognizing and responding to abnormal behaviors or responses during skin-to-skin contact through breastfeeding. Enhancing their awareness and understanding of these interactions can contribute to improved mother-infant emotional bonding and attachment, which are vital aspects of breastfeeding [63]. By addressing these considerations and providing targeted support to parents of ASD children and high-risk parents, we can strive towards optimizing breastfeeding practices and ensuring that the specific needs of these individuals and families are met effectively.

Secondly, healthcare professionals and primary care providers should play a more active role in promoting breastfeeding, particularly when they identify parents with autistic traits during consultations and treatments. It is crucial for these professionals to prioritize breastfeeding support and education in their interactions with these parents. Additionally, incorporating internet-based interventions or online consultations could be a valuable approach to provide continuous support for parents with autistic traits, helping to address any negative experiences they may have encountered with traditional healthcare services, which can often contribute to early breastfeeding cessation [64]. Furthermore, despite a notable increase in exclusive breastfeeding rates among children without ASD, the overall rate remains significantly below the global recommendation [53]. In the US, one of the most common reasons for breastfeeding discontinuation is the need for mothers to return to work or school, which often results in limited time and inadequate facilities for breastfeeding [57]. Therefore, implementing measures to safeguard maternal legal rights, such as extending maternity leave and ensuring the availability of nursing and pumping facilities in public areas, becomes imperative. These actions can actively encourage and enable mothers to continue breastfeeding [65, 66].

### Strengths and limitations

There are limitations to consider in our study. Firstly, as the NSCH is a cross-sectional study, it does not provide evidence of a temporal relationship. However, by combining data from multiple five-year cycles, we aimed to enhance the validity and reliability of our conclusions. Secondly, the survey only collected information on ASD diagnosis and infant feeding practices from respondents with children aged 0 to 5 years. We specifically focused on children between 2 and 5 years of age, as this critical period is known for reliable ASD diagnoses [34]. This age range aligns with the recommendations of the American Academy of Pediatrics for ASD screening [67]. It is worth noting that in children under 2 years old, differentiating ASD symptoms from other developmental disorders can be challenging. Thirdly, it is important to interpret the results with caution and acknowledge the limitations inherent in the data. While weighting helps in mitigating certain biases, it remains essential to carefully consider the sample’s representativeness and potential biases introduced during the weighting process across different years of the NSCH survey [68]. Additionally, the low prevalence of ASD cases included in the logistic regression analysis may result in wide confidence intervals. However, we conducted sensitivity analyses and found consistent results indicating no association, whether considering children with a previous ASD diagnosis or those currently diagnosed with ASD. Fourthly, our study may not have fully adjusted for potential covariates [69, 70].

We lacked data on factors such as the mode of childbirth, timing of ASD diagnosis, maternal medication use, and maternal alcohol and tobacco consumption, which could have influenced the results. These limitations should be taken into account when interpreting our findings. Further research addressing these limitations could provide a more comprehensive understanding of the relationship between infant feeding practices and ASD risk.

## Conclusions

Our study found no significant association between infant feeding practices and ASD among children aged 2–5 years in the US. However, it is important to note that all ORs were consistently in the same direction, indicating a potential trend. The wide confidence intervals observed in our study reflect the limited precision of the estimates due to the relatively low numbers of children with ASD. Therefore, cautious interpretation of the findings is warranted due to several limitations. First, our study design does not provide evidence of a temporal relationship, and the wide confidence intervals further warrant careful interpretation. Additionally, we may not have fully adjusted for potential confounding variables, which could influence the results. Despite the null association, we identified a concerning trend in breastfeeding practices, particularly exclusive breastfeeding, among children with ASD. The rates were far below the optimal levels recommended by health organizations. This highlights the need for targeted policies and practices that address breastfeeding challenges among parents of children with ASD or those at high risk of having a child with ASD. It is crucial for officials and healthcare professionals to prioritize the development of supportive measures, education, and interventions that specifically address the unique needs and circumstances of these parents. By promoting and facilitating breastfeeding in this high-risk population, we can strive to improve infant health outcomes and overall well-being.

### Electronic supplementary material

Below is the link to the electronic supplementary material.

Supplementary Material 1

## Data Availability

The datasets analyzed during the current study are available in the NSCH repository, https://www.childhealthdata.org/.

## Abbreviations

ASD: Autism Spectrum Disorder
DALYs: Disability-adjusted Life Years
NSCH: National Survey of Children’s Health
HRSA: Health Resources and Services Administration
MCHB: Maternal and Child Health Bureau
CAHMI: Child and Adolescent Health Measurement Initiative
DRC: Data Resource Center
PDD: Pervasive Developmental Disorder
FPL: Federal Poverty Level
WHO: World Health Organization
