# Correction: Infant feeding practices and autism spectrum disorder in US children aged 2–5 years: the national survey of children’s health (NSCH) 2016–2020

**DOI:** 10.1186/s13006-023-00595-9

**Published:** 2023-11-13

**Authors:** Xiao-Ling Zhan, Ning Pan, Shamshad Karatela, Lei Shi, Xin Wang, Zhao-Yan Liu, Jin Jing, Xiu-Hong Li, Li Cai, Li-Zi Lin

**Affiliations:** 1 Research Center of Children and Adolescent Psychological and Behavioral Development, Department of Maternal and Child Health, School of Public Health, Sun Yat-sen University, Guangzhou, 510080 China; 2 Key Laboratory of Brain, Cognition and Education Sciences, Ministry of Education; Institute for Brain Research and Rehabilitation, and Guangdong Key Laboratory of Mental Health and Cognitive Science, South China Normal University, Guangzhou, 510631 China; 3 Faculty of Health and Behavioural Sciences, Pharmacy Australia Centre of Excellence, University of Queensland, Woolloongabba, QLD Australia; 4 Institute of Tropical Health and Medicine (AITHM), James Cook University, Townsville, QLD Australia; 5 JNU-HKUST Joint Laboratory for Neuroscience and Innovative Drug Research, College of Pharmacy, Jinan University, Guangzhou, Guangdong 510632 China; 6 Department of Nutrition, School of Public Health, Sun Yat-sen University, Guangzhou, 510080 China; 7 Guangdong Provincial Engineering Technology Research Center of Environmental Pollution and Health Risk Assessment, Department of Occupational and Environmental Health, School of Public Health, Sun Yat-sen University, 74 Zhongshan 2nd Road, Yuexiu District, Guangzhou, 510080 China

**International Breastfeeding Journal (2023) 18:41**

10.1186/s13006-023-00580-2

Following publication of the original article [1], the authors received communication from National Survey of Children’s Health (NSCH) officials regarding a processing error affecting specific variables within the dataset that they had used to investigate the relationship between infant feeding practices and autism spectrum disorder (ASD) among children aged 2–5 years in the United States (US). Following discovery of this error, The Child and Adolescent Health Measurement Initiative (CAHMI) corrected and updated all the affected datasets on its Data Resource Center (DRC) website. Then, following this correction of the datasets, the authors reran their analyses concerning the above-mentioned relationship and made the following adjustments to the article:

1. Among the 472 children diagnosed with ASD, there are 66.6% of them engaging in partial breastfeeding and 10.1% being exclusively breastfed instead of 67.5% and 9.1%, respectively.

2. The adjusted odds ratio (OR) for each additional month of breastfeeding was 0.99 (95% CI, 0.97–1.01) instead of 0.98 (95% CI, 0.96–1.01).

3. When comparing children who never breastfed to those with varying durations of breastfeeding, the adjusted ORs for children breastfed for 0–6 months, 6–12 months, 12–24 months and longer than 24 months were 0.84 (95% CI, 0.51–1.36), 0.76 (95% CI, 0.42–1.35), 0.79 (95% CI, 0.43–1.45), and 0.66 (95% CI, 0.32–1.35) instead of 0.81 (95% CI, 0.50–1.31), 0.65 (95% CI, 0.36–1.18), 0.81 (95% CI, 0.44–1.49), and 0.48 (95% CI, 0.23–1.01), respectively.

4. Compared with children who were never breastfed, the adjusted OR for children who were ever breastfed was 0.79 (95% CI, 0.50–1.25) instead of 0.74 (95% CI, 0.47–1.18).

5. When examining exclusive breastfeeding and partial breastfeeding in relation to no breastfeeding, the adjusted ORs ranged from 1.12 (95% CI, 0.57–2.20) for exclusive breastfeeding to 0.74 (95% CI, 0.47–1.18) for partial breastfeeding instead of 0.96 (95% CI, 0.50–1.85) and 0.72 (95% CI, 0.45–1.15), respectively.

6. Each additional month of breastfeeding was associated with decreased risk of ASD (OR 0.98, 95% CI, 0.97-1.00) in the unweighted analyses instead of 0.98 (95% CI, 0.97–0.99).

7. When comparing children who never breastfed, the adjusted ORs for children breastfed for 6–12 months, ever breastfed and partial breastfeeding were 0.63 (95% CI, 0.48–0.83), 0.81 (95% CI, 0.67-1.00) and 0.80 (95% CI, 0.65–0.98) in the unweighted analyses instead of 0.60 (95% CI, 0.46–0.78), 0.80 (95% CI, 0.66–0.97) and 0.78 (95% CI, 0.64–0.95), respectively.

8. Exclusive breastfeeding for children with ASD decreased from 12.0% in 2016 to 5.9% in 2020 instead of 4.2% in 2020.

The published article has since been corrected. The conclusions of the article are not affected by this change.

The authors thank you for reading this erratum and apologize for any inconvenience caused.
