# Supplementary material for: Infant feeding practices and autism spectrum disorder in US children aged 2–5 years: the national survey of children’s health (NSCH) 2016–2020
Source: Int Breastfeed J. 2023 Aug 11;18:41. doi: 10.1186/s13006-023-00580-2 (PMC10422796; doi:10.1186/s13006-023-00580-2)
Supplement: Supplementary file 1 — Supplementary Material 1 [file 13006_2023_580_MOESM1_ESM.docx]

**Infant feeding Practice and Autism Spectrum Disorders in US Children Aged 2-5 years: the National Survey of Children's Health (NSCH) 2016-2020**

**Xiao-Ling Zhan; Ning Pan; Li Cai; Xin Wang; Xiao-Xuan Ou; Zhao-Yan Liu; Xiu-Hong Li; Jin Jing; Li-Zi Lin**

**Methodology supplement**

**Frame and sample selection**

The NSCH used an address-based sample selected from an extract of the Census Bureau’s Master Address File (MAF), and covers all 50 states as well as the District of Columbia. To ensure a representative sample, the sample frame uses administrative records-based flags to identify three mutually exclusive strata. Stratum 1 contained addresses that are explicitly linked to children using administrative records, with approximately 80% of these addresses being households with children. Stratum 2a contained addresses that are probabilistically linked to children using administrative records and block group characteristics, with approximately 15% of these addresses being households with children. Stratum 2b contained the remaining addresses, with less than 5% of these addresses being households with children.

The sample was selected using a systematic random sampling approach, with the sort order being based on county, poverty, Census tract, Census block, and MAFID within each state and stratum. Sampling intervals determined the households selected to be in sample and were calculated for each of the two sampling strata in each state. The formula was the state-level stratum size on the frame divided by the calculated state-level expected sample size in the stratum. When determining the random start for each stratum of each state, first a uniform random number between (0,1) was generated. The returned value was then multiplied by the sampling interval to get the random start, or the first record to be in sample for that state and stratum.

**Data collection**

The NSCH included a two-phase data collection approach: (1) an initial household screener to assess the presence, basic demographic characteristics, and special health care needs status of any children in the home; and (2) a substantive topical questionnaire to be completed by a parent or caregiver of the selected child. The data collection methodology employed strategies to increase response, including clear and concise question wording, providing response mode options, cash incentives and other treatments.

The survey was administered both online and by mail. Randomly selected addresses from households across the United States were mailed instructions to access the online survey, and some addresses also received a paper version of the screening questionnaire. After two reminder letters and postcard reminders to complete the survey by web, those households who had not accessed the online survey were mailed a paper screening questionnaire. This methodology was designed to ensure a high response rate and accurate representation of the health and well-being of children in the United States.

**Data collection results**

**2016:** Of the sampled addresses, a total of 139,923 households in the 50 states and the District of Columbia were screened for age-eligible children. Of these households, 68,961 reported age-eligible children living or staying in the household. From this sample of households with age-eligible children, 50,212 detailed topical questionnaires were completed.

**2017:** Of the sampled addresses, a total of 59,135 households in the 50 states and the District of Columbia were screened for age-eligible children. Of these households, 29,968 reported age-eligible children living or

staying in the household. From the sample of households with age-eligible children, 21,599 detailed topical questionnaires were completed.

**2018:** Of the 176,052 sampled addresses in the 50 states and the District of Columbia, 40.2% were screened for age-eligible children. Of these households, 55.1% reported age-eligible children living or staying in the household. 38,140 screener questionnaires with children were completed. From the sample of households with age-eligible children, 30,530 detailed topical questionnaires were completed.

**2019:** Of the approximately 180,000 sampled addresses in the 50 states and the District of Columbia, 38.1% were screened for age-eligible children. Of these households, 54.0% reported age-eligible children living or staying in the household. 36,196 screener questionnaires with children were completed. From the sample of households with age-eligible children, 29,433 detailed topical questionnaires were completed.

**2020:** Of the approximately 240,000 sampled addresses in the 50 states and the District of Columbia, 39.1% were screened for age-eligible children. Of these households, 55.8% reported age-eligible children living or staying in the household. 51,107 screener questionnaires with children were completed. From the sample of households with age-eligible children, 42,777 detailed topical questionnaires were completed.

.

Table S1 Overview of the studies about the association between breastfeeding and risk of ASD

| Author | Country | Study Design | Sample size | Age | Outcome definition | | Exposure definition  Breastfeeding categories |  | Matched/Adjusted Factors if Present |
| --- | --- | --- | --- | --- | --- | --- | --- | --- | --- |
|  |  |  |  |  | Diagnostic procedure | Diagnostic Method |  |  |  |
| Bittker, et al.(2018)^1^ | USA | Case-control | 1001 ASD 514 Controls | 3-12 years | Parent-report | NA | Breastfeeding duration(months) | 0.954  (0.935-0.973) | age, ethnicity, maternal education, Midwest and south |
| Manohar, et al. (2018)^2^ | India | Case-control | 30 ASD 30 TD siblings | 2-6 years | CARS | DSM-5 | Received exclusive breastfeeding up to 6 months or not | 0.166  (0.025–0.65) | Not reported |
| George, et al. (2014)^3^ | India | Case-control | 143 ASD 200 Controls | 2-6 years | CARS | NA | Breastfeeding duration nil/ less than 6 month or not | 3.4  (1.28-8.99) | Not reported |
| Brown, et al. (2014)^4^ | Australia | Case-control | 19 ASD 23 TD siblings | NA | Administrative databases | NA | First hour breastfeeding or not | 0.26  (0.07-0.89) | Not reported |
| AI-Farsi, et al. (2012)^5^ | Oman | Case-control | 102 ASD 102 Controls | 3-14 years | CARS | DSM-Ⅳ-TR | First hour breastfeeding or not  Exclusive breastfeeding duration in categories  (<3 months, ≤ 3 months, ≤ 4 months, ≤ 5 months and ≤ 6 months)  Continued breast-feeding in categories (<1 month, 1–6 months, 7–12 months and 13–18 months with 19–24 months) | 1.48  (1.01-3.1)  3.3  (0.29–38),  2.97  (1.62–5.5)  0.99  (0.33–2.99)  0.41  (0.04–3.87)  6.6 (1.9–22.3) 3.79 (1.5–9.5) 3.52 (1.42–8.8)  2.14 (0.9–5.1) | Maternal age, maternal education, family income, and antenatal and perinatal complications |
| Bawono, et al. (2012)^6^ | Indonesia | Case-control | 52 ASD 104 Controls | 3-10 years | Diagnostic procedure by an expert child and adolescent psychiatrist | DSM-Ⅳ-TR | Breastfeeding ≥ 6 months vs never breastfeeding  Breastfeeding duration in categories (never exclusive breastfeeding, 0–6 months, 6-<12 months and ≥ 12 months) | 2.01 (1.02-3.96) 5.75 (1.4-23.49) 2.17 (0.73-6.46) 1.59 (0.54-4.62) | Child gender, age, and residence |
| Schultz, et al. (2006)^7^ | USA | Case-control | 861 ASD 123 Controls | 2-18 years | Parent-report | NA | Breastfeeding duration in categories  (never, < 2 months, 2–6 months, and ≥ 6 months) | 2.48 (1.42-4.35)  1.70 (1.00-2.88)  1.27 (0.75-2.14)  0.69 (0.32–1.48), 0.51 (0.24–1.1), and 0.4 (0.230.71) | Age |
| Lemcke, et al. (2018)^8^ | Denmark | Cohort | 76322 with 973 ASD 300 ID | Mean age = 11.3 | NA | ICD-10 | Breastfeeding duration in categories  (≤6 months, 7-10 months, ≥ 11 months) | 0.8 (0.7,1.0)  0.7(0.6,0.8) | Not reported |
| Kim, et al. (2021)^9^ | Korea | Cross-sectional | 374074 with 606 ASD | 6 months-10 years of age | Administrative databases | ICD-10 | Breastfeeding during the first 4 to 6 months or not | 0.72 (0.57-0.89) | Socioeconomic status and perinatal factors |
| Chen, et al. (2021)^10^ | China | Cross-sectional | 67578 with 2074 autistic trait | 4.61±0.88 years | ABC | Undiagnosed | Breastfeeding or not  Maternal Participation in Prenatal Education | 0.773 (0.676–0.884) | child’s age, child’s gender, parental education level, parents’ age at child’s birth, monthly family income and parental marital status |
| Soke, et al. (2019)^11^ | USA | Cross-sectional | 673 ASD 876 Controls | 30-68 months | ADOS, ADI-R | DSM-Ⅳ | Breastfeeding duration in categories (high tertile (12 months) with low (<6 months) or the middle (6-<12 months) | 0.61(0.45-0.84) 0.72 (0.54-0.98) | child sex, gestational age, birth weight, 5-min Apgar score, birth plurality, mode of delivery; mother’s age at the time of child’s birth in years, parity with the index child, race and ethnicity, education at time of child’s birth, place of birth, employment status during the 3 months before the child’s birth until the end of breastfeeding period, marital status, smoking status, and presence of neuropsychiatric diagnoses; family estimated income during the year preceding the index child’s birth, and study site |
| Huang, et al. (2021)^12^ | China | Cross-sectional | 6049 with 71 ASD | 16–30 months | M-CHAT-R/F  CARS | DSM-5 | Breastfeeding status (exclusive breastfeeding, partial breastfeeding, not breastfeeding) | 1.55 (0.90, 2.74)  2.34 (1.10, 4.82) | child’s age, sex, only child, maternal age, maternal education level, yearly household income, ethnic background and study area, preterm birth, pregnancy information (complications and depression during pregnancy), second-hand smoke exposure and overweight/obesity before pregnancy. |
| Ravi, et al. (2016)^13^ | India | Cross-sectional | 33 High-risk ASD  36 Medium-risk ASD  281 Low-risk ASD | 16-30 months | M-CHAT-R | Undiagnosed | Initiation breastfeeding or not  Exclusive breastfeeding in the first 6 months or not | 3.9 (1.83-8.39) 0.2 (0.05-0.91) | Not reported |
| Berding, et al. (2018)^14^ | Cham-paign Urbana | Case-control | 26 ASD  32 Controls | 2-7 years | NA | NA | Rate of early feeding mode (breast-fed only, breast-fed in combination with formula, formula only) | ASD:20%, 65%, 15%  Controls: 53%, 41%, 6% | Age- and sex-matched |
| Shafai, et al. (2014)^15^ | USA | Case-control | 60 ASD  85 Controls | ND | Parent-report | NA | Breastfeeding duration in categories  (< 2 months, 2–4 months, 4–6 months, 6–9 months, 9–12 months, 12–15 months, 15–18 months, 18–24 months, and ≥ 24 months) | Not reported | Not reported |
| Boucher, et al. (2017)^16^ | Spanish | Cross-sectional | Autistic trait | 4-6.9 year | CAST | Undiagnosed | Ever-breastfeeding duration (months)  Predominant breastfeeding duration (months)  Exclusive breastfeeding duration (months) | 1.00(0.95,1.05) 0.98 (0.87,1.10) 1.02 (0.86-1.22) | child care, maternal variables (parity at birth, age, education, social status, country of birth, smoking during pregnancy, verbal IQ proxy, psychopathology), and paternal variables (education and social class). |
| Husk, et al. (2015)^17^ | USA | Cross-sectional | 391 ASD 37901 Controls | 2-5years | Parent-report | DSM-Ⅳ | Ever-breastfeeding or not  3-month exclusive breastfeeding in categories (never, at least one, at least 3 months)  6-month exclusive breastfeeding in categories (never, at least one, at least 6 months) | 0.7 (0.4, 1.3)  0.7 (0.4–1.5)  0.6 (0.3–1.3)  0.7 (0.3–1.7)  0.7 (0.3–1.3) | Child gender, age, race, birth order, parental education, mother’s age, family income, and whether the child had a personal doctor or nurse |
| Whitely, et al. (2021)^18^ | Australia | Cross-sectional | 293 ASD  3158 controls | 7.3±2.7  6.3±2.8 | Parent-report | NA | Breastfed exclusively | 0.73 (0.52–1.01) | Mother education level, mother ASD, father ASD, child age, child ADHD, child intellectual disability |
| Kara, et al. (2020)^19^ | Turkey | Case-control | 141 ASD  128 Controls | 24–72 months | NA | DSM-5 | Breastfeeding started immediately  Total duration of breastfeeding (month)  Exclusive breastfeeding (month) | 1.508 (0.735–3.097)  0.987 (0.962–1.013)  0.936 (0.839–1.045) | sex of child |
| Say, et al. (2016)^20^ | Turkey | Case-control | 100 ASD  100 ADHD  80 Controls | 3-18 years | Diagnostic procedure by an expert child and adolescent psychiatrist | DSM-Ⅳ | Breastfeeding duration (month) | Not reported | Age and gender-matched |
| Field, et al. (2014)^21^ | USA | Case-control | 31 ASD  81 ADHD 612 Controls | NA | Diagnostic procedure by an expert child and adolescent psychiatrist | DSM-Ⅳ-TR | Breastfeeding at least 4 months or not | Not reported | Age/sex-matched |
| Soke, et al. (2019)^11^ | USA | Cross-sectional | 673 ASD 876 Controls | 30-68 months | ADOS, ADI-R | DSM-Ⅳ | Breastfeeding initiation or not | 0.88 (0.60-1.28) | child sex, gestational age, birth weight, 5-min Apgar score, birth plurality, mode of delivery; mother’s age at the time of child’s birth in years, parity with the index child, race and ethnicity, education at time of child’s birth, place of birth, employment status during the 3 months before the child’s birth until the end of breastfeeding period, marital status, smoking status, and presence of neuropsychiatric diagnoses; family estimated income during the year preceding the index child’s birth, and study site |
| Emond, et al. (2010)^22^ | England | Cohort | 79 ASD  12901 Controls | 0-5 years | a multidisciplinary assessment | NA | Rate of breastfeeding | Not reported | Not reported |
| Burd, et al. (1988)^23^ | USA | Case-control | 50 PDD  50 Controls | 9.0±4.6 years 8.8±4.7 years | Diagnostic procedure by an expert child and adolescent psychiatrist | DSM-Ⅲ | Rate of breastfeeding  Reason given for not breastfeeding | Not reported | Age, sex and IQ |
| Tanoue, et al. (1989)^24^ | Japan | Case-control | 145 ASD  224 Controls | 3 years | Diagnostic procedure by an expert child and adolescent psychiatrist | DSM-Ⅲ | Breastfeeding duration in categories  (< 1 week, 1 week - 1 month, 1–2 months, 2–3 months, 3–6 months, 6–12 months, and ≥ 12 months) | Not reported | Not reported |
| Dodds, et al. (2011)^25^ | Canada | Cohort | 129733 with 924 ASD | 1-17 years | Administrative databases | ICD-9 or ICD 10 | Breastfeeding at discharge or not | 1.15 (1.01-1.31) | Birth year and genetic susceptibility (defined as having a sibling with autism or a mother with an underlying psychiatric or neurologic illness) |
| Shamberger, et al. (2011)^26^ | USA | Ecological study | ASD Controls | 3–21 years | NA | NA | Rate of breastfeeding | Not reported | Not reported |

**Abbreviations:** ASD, autism spectrum disorder; DSM- 5, Diagnostic and Statistical Manual of Mental Disorders, Fifth Edition; DSM-Ⅳ-TR, the text revision of the fourth edition of the Diagnostic and Statistical Manual of Mental Disorders; DSM-Ⅳ, Diagnostic and Statistical Manual of Mental Disorders, Fourth Edition; DSM-Ⅲ, Diagnostic and Statistical Manual of Mental Disorders, Third Edition; ICD-9, International Classification of Diseases, Ninth Revision; ICD-10, International Classification of Diseases, 10th Revision; CARS, Childhood Autism Rating Scale; TD, typically development; ABC, Autism Behavior Checklist; ADI-R, Autism Diagnostic Interview-Revised; CAST, Childhood Autism Spectrum Test; M-CHAT-R, Modified Checklist of Autism in Toddlers, Revised;

Table S2. Associations of breastfeeding practice with ASD among US children aged 2-5 years by sex

|  | **Male** | | | |  | **Female** | | | |
| --- | --- | --- | --- | --- | --- | --- | --- | --- | --- |
|  | ***t* value** | **df** | **OR (95% CI)** | ***P* value** |  | ***t* value** | **df** | **OR (95% CI)** | ***P* value** |
| **Breastfeeding Duration, Months** | -0.36 | 17701 | 1.00 (0.97, 1.02) | 0.72 |  | -1.04 | 16455 | 0.97 (0.91, 1.03) | 0.30 |
|  |  |  |  |  |  |  |  |  |  |
| **Breastfeeding Duration in Categories** | |  |  |  |  |  |  |  |  |
| **Never** | *Reference* | | | |  | *Reference* | | | |
| **>0-6 months** | -0.22 | 17698 | 0.94 (0.56, 1.60) | 0.83 |  | -1.00 | 16452 | 0.60 (0.22, 1.62) | 0.32 |
| **>6-12 months** | -0.51 | 17698 | 0.84 (0.44, 1.62) | 0.61 |  | -1.04 | 16452 | 0.54 (0.17, 1.73) | 0.30 |
| **>12-24 months** | 0.03 | 17698 | 1.01 (0.53, 1.94) | 0.98 |  | -1.37 | 16452 | 0.36 (0.08, 1.55) | 0.17 |
| **>24 months** | -1.40 | 17698 | 0.55 (0.24, 1.27) | 0.16 |  | -0.05 | 16452 | 0.97 (0.25, 3.69) | 0.96 |
|  |  |  |  |  |  |  |  |  |  |
| **Ever Breast-fed** |  |  |  |  |  |  |  |  |  |
| **No** | *Reference* | | | |  | *Reference* | | | |
| **Yes** | -0.43 | 18087 | 0.90 (0.55, 1.47) | 0.67 |  | -1.20 | 16821 | 0.55 (0.21, 1.46) | 0.23 |
|  |  |  |  |  |  |  |  |  |  |
| **Breastfeeding Status** |  |  |  |  |  |  |  |  |  |
| **No Breastfeeding** | *Reference* | | | |  | *Reference* | | | |
| **Partial Breastfeeding** | -0.62 | 18086 | 0.86 (0.52, 1.40) | 0.54 |  | -1.43 | 16820 | 0.49 (0.18, 1.30) | 0.15 |
| **Exclusive Breastfeeding** | 0.51 | 18086 | 1.22 (0.57, 2.62) | 0.61 |  | -0.13 | 16820 | 0.92 (0.26, 3.31) | 0.90 |
| All models were adjusted for child age, child sex, year of birth, child race, family income, and birth order. | | | | | | | | | |

Table S3. Associations of breastfeeding practice with current ASD among US children aged 2-5 years by sex

|  | **Male** | | | |  | **Female** | | | |
| --- | --- | --- | --- | --- | --- | --- | --- | --- | --- |
|  | ***t* value** | **df** | **OR (95% CI)** | ***P* value** |  | ***t* value** | **df** | **OR (95% CI)** | ***P* value** |
| **Breastfeeding Duration, Months** | -0.21 | 17695 | 1.00 (0.97, 1.02) | 0.83 |  | -1.04 | 16455 | 0.97 (0.91, 1.03) | 0.30 |
|  |  |  |  |  |  |  |  |  |  |
| **Breastfeeding Duration in Categories** | |  |  |  |  |  |  |  |  |
| **Never** | *Reference* | | | |  | *Reference* | | | |
| **>0-6 months** | 0.38 | 17692 | 1.10 (0.66, 1.84) | 0.70 |  | -1.15 | 16452 | 0.55 (0.20, 1.52) | 0.25 |
| **>6-12 months** | -0.02 | 17692 | 0.99 (0.51, 1.92) | 0.98 |  | -1.04 | 16452 | 0.53 (0.16, 1.75) | 0.30 |
| **>12-24 months** | 0.47 | 17692 | 1.17 (0.60, 2.29) | 0.64 |  | -1.39 | 16452 | 0.33 (0.07, 1.56) | 0.16 |
| **>24 months** | -1.54 | 17692 | 0.49 (0.19, 1.22) | 0.12 |  | 0.02 | 16452 | 1.01 (0.26, 3.88) | 0.99 |
|  |  |  |  |  |  |  |  |  |  |
| **Ever Breast-fed** |  |  |  |  |  |  |  |  |  |
| **No** | *Reference* | | | |  | *Reference* | | | |
| **Yes** | 0.17 | 18081 | 1.04 (0.64, 1.68) | 0.87 |  | -1.29 | 16820 | 0.52 (0.19, 1.41) | 0.20 |
|  |  |  |  |  |  |  |  |  |  |
| **Breastfeeding Status** |  |  |  |  |  |  |  |  |  |
| **No Breastfeeding** | *Reference* | | | |  | *Reference* | | | |
| **Partial Breastfeeding** | -0.05 | 18080 | 0.99 (0.61, 1.59) | 0.96 |  | -1.54 | 16819 | 0.46 (0.17, 1.23) | 0.12 |
| **Exclusive Breastfeeding** | 0.99 | 18080 | 1.47 (0.68, 3.17) | 0.32 |  | -0.13 | 16819 | 0.92 (0.25, 3.32) | 0.90 |
| All models were adjusted for child age, child sex, year of birth, child race, family income, and birth order. | | | | | | | | | |

Table S4. Testing for linear trends of Breastfeeding practice in all participants aged 2-5 years from 2016 to 2020

|  | **Exclusive breastfeeding** | | |  | **Ever breastfeeding** | | | |
| --- | --- | --- | --- | --- | --- | --- | --- | --- |
|  | **Model 1** | **Model 2** | **Model 3** |  | **Model 1** | **Model 2** | **Model 3** | |
| (Intercept) | -2.64 (0.28) ^**^ | -2.64 (0.28) ^**^ | -2.64 (0.28) ^**^ |  | 0.75 (0.23) ^**^ | 0.75 (0.23) ^**^ | 0.75 (0.23) ^**^ | |
| Linear contrast value | 0.28 (0.09) ^***^ | 0.28 (0.08) ^***^ | 0.28 (0.08) ^***^ |  | 0.15 (0.07) ^*^ | 0.15 (0.07) ^*^ | 0.15 (0.07) ^*^ | |
| Quadratic contrast value |  | 0.00 (0.09) | -0.00 (0.09) |  |  | -0.04 (0.07) | -0.04 (0.07) | |
| Cubic contrast value |  |  | 0.10 (0.09) |  |  |  | 0.01 (0.08) | |
| Deviance | 22329.11 | 22329.10 | 22322.66 |  | 32588.16 | 32586.70 | 32586.60 | |
| Dispersion | 1.00 | 1.00 | 1.00 |  | 0.99 | 0.99 | 0.99 | |
| Num. obs. | 35050 | 35050 | 35050 |  | 35050 | 35050 | 35050 | |
| ^***^*P* value < 0.001; ^**^ *P* value < 0.01; ^*^ *P* value < 0.05  All models were adjusted for child age, child sex, year of birth, child race, family income, and birth order. | | | | | | | |  |

Table S5. Testing for linear trends of Breastfeeding practice in children without ASD aged 2-5 years from 2016 to 2020

|  | **Exclusive breastfeeding** | | |  | **Ever breastfeeding** | | |
| --- | --- | --- | --- | --- | --- | --- | --- |
|  | **Model 1** | **Model 2** | **Model 3** |  | **Model 1** | **Model 2** | **Model 3** |
| (Intercept) | -2.59 (0.29) ^***^ | -2.59 (0.28) ^***^ | -2.59 (0.28) ^***^ |  | 0.76 (0.24) ^**^ | 0.76 (0.24) ^**^ | 0.76 (0.24) ^**^ |
| Linear contrast value | 0.30 (0.09) ^***^ | 0.30 (0.08) ^***^ | 0.30 (0.08) ^***^ |  | 0.16 (0.07) ^*^ | 0.16 (0.07) ^*^ | 0.16 (0.07) ^*^ |
| Quadratic contrast value |  | 0.01 (0.09) | 0.00 (0.09) |  |  | -0.02 (0.07) | -0.02 (0.07) |
| Cubic contrast value |  |  | 0.10 (0.09) |  |  |  | 0.00 (0.08) |
| Deviance | 21859.74 | 21859.68 | 21853.27 |  | 31827.12 | 31826.62 | 31826.61 |
| Dispersion | 1.00 | 1.00 | 1.00 |  | 1.00 | 1.00 | 1.00 |
| Num. obs. | 34434 | 34434 | 34434 |  | 34434 | 34434 | 34434 |
| ^***^*P* value < 0.001; ^**^ *P* value < 0.01; ^*^ *P* value < 0.05  All models were adjusted for child age, child sex, year of birth, child race, family income, and birth order. | | | | | | | |

Table S6. Testing for linear trends of Breastfeeding practice in children with ASD aged 2-5 years from 2016 to 2020

|  | **Exclusive breastfeeding** | | |  | **Ever breastfeeding** | | |
| --- | --- | --- | --- | --- | --- | --- | --- |
|  | **Model 1** | **Model 2** | **Model 3** |  | **Model 1** | **Model 2** | **Model 3** |
| (Intercept) | -4.65 (1.70) ^**^ | -4.49 (1.69) ^**^ | -4.56 (1.74) ^**^ |  | -1.06 (1.12) | -0.98 (1.10) | -1.07 (1.11) |
| Linear contrast value | -0.63 (0.51) | -0.70 (0.56) | -0.69 (0.55) |  | 0.08 (0.40) | 0.09 (0.39) | 0.07 (0.38) |
| Quadratic contrast value |  | -0.44 (0.48) | -0.43 (0.47) |  |  | -0.32 (0.41) | -0.36 (0.42) |
| Cubic contrast value |  |  | 0.28 (0.55) |  |  |  | 0.34 (0.41) |
| Deviance | 375.12 | 373.39 | 372.63 |  | 602.41 | 600.66 | 598.68 |
| Dispersion | 1.31 | 1.36 | 1.36 |  | 1.13 | 1.13 | 1.13 |
| Num. obs. | 616 | 616 | 616 |  | 616 | 616 | 616 |
| ^***^*P* value < 0.001; ^**^ *P* value < 0.01; ^*^ *P* value < 0.05  All models were adjusted for child age, child sex, year of birth, child race, family income, and birth order. | | | | | | | |

1. Bittker SS, Bell KR. Acetaminophen, antibiotics, ear infection, breastfeeding, vitamin D drops, and autism: an epidemiological study. *Neuropsychiatric disease and treatment.* 2018;14:1399-1414.

2. Manohar H, Pravallika, M., Kandasamy, P., Chandrasekaran, V., & Rajkumar, R. P. . Role of Exclusive Breastfeeding in Conferring Protection in Children At-Risk for Autism Spectrum Disorder: Results from a Sibling Case-control Study. *Journal of neurosciences in rural practice.* 2018;9(1):132-136.

3. George B, Padmam MS, Nair MK, Leena ML, Russell PS. CDC Kerala 14: Early child care practices at home among children (2-6 y) with autism--a case control study. *Indian J Pediatr.* 2014;81 Suppl 2:S138-141.

4. Brown CM, Austin DW, Busija L. Observable essential fatty acid deficiency markers and autism spectrum disorder. *Breastfeed Rev.* 2014;22(2):21-26.

5. Al-Farsi YM, Al-Sharbati MM, Waly MI, et al. Effect of suboptimal breast-feeding on occurrence of autism: a case-control study. *Nutrition (Burbank, Los Angeles County, Calif).* 2012;28(7-8):e27-32.

6. Bawono KDH, E.S.; Wandita S. Breastfeeding as a protective factor against autism. *JURNAL GIZI KLINIK INDONESIA.* 2012;8(4):166-171.

7. Schultz ST, Klonoff-Cohen HS, Wingard DL, et al. Breastfeeding, infant formula supplementation, and Autistic Disorder: the results of a parent survey. *International breastfeeding journal.* 2006;1:16.

8. Lemcke S, Parner ET, Bjerrum M, Thomsen PH, Lauritsen MB. Early Regulation in Children Who Are Later Diagnosed with Autism Spectrum Disorder. A Longitudinal Study within the Danish National Birth Cohort. *Infant Ment Health J.* 2018;39(2):170-182.

9. Kim JH, Lee SW, Lee JE, Ha EK, Han MY, Lee E. Breastmilk Feeding during the First 4 to 6 Months of Age and Childhood Disease Burden until 10 Years of Age. *Nutrients.* 2021;13(8).

10. Chen J, Strodl E, Huang LH, et al. Associations between Prenatal Education, Breastfeeding and Autistic-Like Behaviors in Pre-Schoolers. *Children (Basel).* 2021;8(2).

11. Soke GN, Maenner M, Windham G, et al. Association Between Breastfeeding Initiation and Duration and Autism Spectrum Disorder in Preschool Children Enrolled in the Study to Explore Early Development. *Autism research : official journal of the International Society for Autism Research.* 2019;12(5):816-829.

12. Huang S, Wang X, Sun T, et al. Association of Breastfeeding for the First Six Months of Life and Autism Spectrum Disorders: A National Multi-Center Study in China. *Nutrients.* 2021;14(1).

13. Ravi S, Chandrasekaran V, Kattimani S, Subramanian M. Maternal and birth risk factors for children screening positive for autism spectrum disorders on M-CHAT-R. *Asian journal of psychiatry.* 2016;22:17-21.

14. Berding K, Donovan SM. Diet Can Impact Microbiota Composition in Children With Autism Spectrum Disorder. *Front Neurosci.* 2018;12:515.

15. Shafai T, Mustafa M, Hild T, Mulari J, Curtis A. The association of early weaning and formula feeding with autism spectrum disorders. *Breastfeed Med.* 2014;9(5):275-276.

16. Boucher O, Julvez J, Guxens M, et al. Association between breastfeeding duration and cognitive development, autistic traits and ADHD symptoms: a multicenter study in Spain. *Pediatric research.* 2017;81(3):434-442.

17. Husk JS, Keim SA. Breastfeeding and Autism Spectrum Disorder in the National Survey of Children's Health. *Epidemiology (Cambridge, Mass).* 2015;26(4):451-457.

18. Whitely A, Shandley K, Huynh M, Brown CM, Austin DW, Bhowmik J. Brief Report: Pregnancy, Birth and Infant Feeding Practices: A Survey-Based Investigation into Risk Factors for Autism Spectrum Disorder. *Journal of autism and developmental disorders.* 2021.

19. Kara T, Alpgan Ö. Nursing personality and features in children with autism spectrum disorder aged 0-2: an exploratory case-control study. *Nutritional neuroscience.* 2020:1-9.

20. Say GN, Karabekiroglu K, Babadagi Z, Yuce M. Maternal stress and perinatal features in autism and attention deficit/hyperactivity disorder. *Pediatr Int.* 2016;58(4):265-269.

21. Field SS. Interaction of genes and nutritional factors in the etiology of autism and attention deficit/hyperactivity disorders: a case control study. *Med Hypotheses.* 2014;82(6):654-661.

22. Emond A, Emmett P, Steer C, Golding J. Feeding symptoms, dietary patterns, and growth in young children with autism spectrum disorders. *Pediatrics.* 2010;126(2):e337-342.

23. Burd L, Fisher W, Kerbeshian J, Vesely B, Durgin B, Reep P. A comparison of breastfeeding rates among children with pervasive developmental disorder, and controls. *Journal of developmental and behavioral pediatrics : JDBP.* 1988;9(5):247-251.

24. Tanoue Y, Oda S. Weaning time of children with infantile autism. *Journal of autism and developmental disorders.* 1989;19(3):425-434.

25. Dodds L, Fell DB, Shea S, Armson BA, Allen AC, Bryson S. The role of prenatal, obstetric and neonatal factors in the development of autism. *Journal of autism and developmental disorders.* 2011;41(7):891-902.

26. Shamberger RJ. Autism rates associated with nutrition and the WIC program. *J Am Coll Nutr.* 2011;30(5):348-353.
